# Effect of annealing treatments on CeO_2_ grown on TiN and Si substrates by atomic layer deposition

**DOI:** 10.3762/bjnano.9.83

**Published:** 2018-03-15

**Authors:** Silvia Vangelista, Rossella Piagge, Satu Ek, Alessio Lamperti

**Affiliations:** 1 CNR-IMM, Unit of Agrate Brianza, Via C. Olivetti 2, Agrate Brianza (MB) I-20864 Italy; 2 STMicroelectronics, Via C. Olivetti 2, Agrate Brianza (MB) I-20864 Italy; 3 Picosun Oy, Tietotie 3, Espoo FI-02150 Finland

**Keywords:** atomic layer deposition, cerium(IV) oxide (CeO_2_) microstructure, in situ annealing, transmission electron microscopy, X-ray diffraction

## Abstract

In this work, we investigate the effect of thermal treatment on CeO_2_ films fabricated by using atomic layer deposition (ALD) on titanium nitride (TiN) or on silicon (Si) substrates. In particular, we report on the structural, chemical and morphological properties of 25 nm thick ceria oxide with particular attention to the interface with the substrate. The annealing treatments have been performed in situ during the acquisition of X-Ray diffraction patterns to monitor the structural changes in the film. We find that ceria film is thermally stable up to annealing temperatures of 900 °C required for the complete crystallization. When ceria is deposited on TiN, the temperature has to be limited to 600 °C due to the thermal instability of the underlying TiN substrate with a broadening of the interface, while there are no changes detected inside the CeO_2_ films. As-deposited CeO_2_ films show a cubic fluorite polycrystalline structure with texturing. Further, after annealing at 900 °C an increase of grain dimensions and an enhanced preferential (200) orientation are evidenced. These findings are a strong indication that the texturing is an intrinsic property of the system more than a metastable condition due to the ALD deposition process. This result is interpreted in the light of the contributions of different energy components (surface energy and elastic modulus) which act dependently on the substrate properties, such as its nature and structure.

## Introduction

Cerium dioxide (CeO_2_) has raised renewed interest in the recent years in many fields of research thanks to its properties, such as the ability to store and release oxygen, or its chemical stability [1–2]. For many applications, this material is useful when synthesized as nanoparticles [3]. However, for specific fields, such as microelectronics and optics, its deposition as thin film highlighted some interesting physical properties, such as the lattice constant close to the value of Si (*a* = 0.541 nm) [4] and the permittivity (κ) of 23–24 [5]. In all the cases, the valence of the Ce ions is fundamental, since it determines some of the material specific ability, such as the oxygen storage. More generally, the structure of cerium oxide is essential in defining its specific capabilities [6]. At the thermodynamic equilibrium under standard conditions of temperature and pressure, stoichiometric CeO_2_ hosts Ce^4+^ and O^2−^ ions in the cubic fluorite structure (space group *Fm−3m* in Pearson notation). However, Ce^3+^ ions can also be accommodated in the lattice structure in relation with oxygen vacancies, and Ce_2_O_3_ as minority phase or sub-stoichiometric CeO_2_ (CeO_2−δ_) can easily form. The cerium ion valence and the ceria structure can be influenced by the substrate onto which the film is grown and by thermal treatment after its deposition [7]. Also, the structural and/or the chemical composition of the substrate can influence the preferential orientation of ceria [8–11]. Further, the specific conditions of the thermal treatment (i.e., high or low temperature in reactive (O_2_) or inert (N_2_) atmosphere) can influence the formation of defects in the crystalline structure, such as grain boundaries, the crystallite growth and the oxygen vacancies, while preserving the cubic fluorite structure [12–14]. The electrical properties of CeO_2_ have been improved by performing post-deposition annealing at various temperatures [15]. In light of these previous studies, the interaction between substrate and annealing is intriguing and could be considered as a valuable approach for fine-tuning the properties of ceria.

Extending the results of our recent study on as-deposited CeO_2_ films on Si and on TiN substrates [8], this work aims to show the structural and chemical properties of CeO_2_ films deposited on the same substrates when annealed at different temperatures and in reactive or inert gas atmospheres. A set of samples has been annealed at a temperature slightly above the growth temperature (300 °C), a value compatible with a back-end process flow typically used in microelectronics CMOS integration process, and in O_2_ atmosphere with the aim to passivate the film defects, namely the oxygen vacancies. A second set of samples has been annealed at high temperatures (600–900 °C) to induce full crystallization in CeO_2_ films and follow the corresponding structural and chemical changes induced by the annealing process. Incidentally, such a high-temperature regime is typical of front-end CMOS processes, and could be of relevance to evaluate a possible CeO_2_ integration at this process step.

Deposition of CeO_2_ thin films in previous studies has been achieved by using a variety of growth techniques, such as RF-magnetron sputtering [7], e-beam [16], physical vapor deposition [17], chemical vapor deposition (CVD) [18], and atomic layer deposition (ALD). The latter has been explored by using different precursors, e.g., Ce(thd)_4_, Ce(iPrCp)_3_ and Ce(mmp)_4_) [19–23], obtaining as-deposited film with polycrystalline structure [8,16–18], which influences the dielectric behavior [18]. ALD guarantees high compatibility with many applications, thanks to the atomic level control of the film thickness with large-area uniformity and conformality also on 3D surfaces. In this study, we use Ce β-diketonate as metal source since its well-established chemistry and the low level of impurities incorporated during growth [16].

## Experimental

Cerium dioxide thin films were deposited in a Picosun R-200 Advanced ALD reactor by Picosun Oy at 250 °C on both Si and TiN-coated 7 × 7 cm^2^ Si substrates simultaneously, by placing them on an 8″ silicon wafer, which was put in the wafer holder. The details of the deposition process can be found in [8]. Some samples have been annealed immediately after the deposition in the reactor chamber without vacuum breaking at a temperature of 300 °C and in oxygen atmosphere for 600 s.

The deposited structures have been characterized by X-ray reflectivity (XRR) and diffraction at grazing incidence (GIXRD) using a XRD3000 diffractometer (Italstructure) with monochromated X-ray Cu Kα radiation (wavelength 0.154 nm) and beam size defined by slits aperture of 0.1 × 6 mm. The high temperature annealing has been performed by using an Anton Paar tool mounted in the diffractometer. This allows us to collect the X-ray diffraction profile during annealing to monitor the evolution of crystallinity of the films during the thermal treatment. During the annealing the sample was kept in static N_2_ atmosphere by using a graphite dome. Thickness, interface and surface roughness, and electronic density have been determined by data fitting using the MAUD software program [24]. MAUD has also been used for Rietveld refinement of GIXRD patterns, allowing to determine CeO_2_ cell parameter and crystallite size [25].

Focused ion beam has been used to prepare lamellae of selected samples for transmission electron microscopy (TEM) analysis (cross and plan view, Fei Tecnai G2) in bright or dark field at a voltage of 200 kV. Plan view images are treated with free software to obtain the crystallite size distribution as in [26].

Time-of-flight secondary ion mass spectrometry (ToF-SIMS) investigation of the compositional depth profile of the CeO_2_/TiN/Si and CeO_2_/Si structures has been performed by means of a Cs^+^ ion beam (energy of 0.5 keV, ion current 38.0 nA) sputtering a 200 μm × 200 μm area, and a Ga^+^ ion beam (25 keV, 2.6 pA) for analysis over a 50 μm × 50 μm area centered on the sputtered crater and therein collecting secondary negative ions [27]. The recorded intensities of the secondary ions were normalized to the intensity of ^30^Si in bulk Si.

The chemical state of the annealed CeO_2_ films [28] was obtained by using X-ray photoelectron spectroscopy (XPS) measurements performed on a PHI 5600 instrument (Physics Electronics Inc.) equipped with a monochromatic Al Kα X-ray source (energy = 1486.6 eV) and a concentric hemispherical analyzer. The spectra were collected at a take-off angle of 45° and a band-pass energy of 23.50 eV. The instrument resolution is 0.5 eV. The spectra were aligned using the C 1s peak (284.6 eV) as reference [29]. The experimental data were fitted with Gaussian–Lorentzian peaks and Shirley background by using the XPSPeak software program (version 4.1, Freeware, University of Warwick, United Kingdom). The spin–orbit splitting and doublet intensity ratio have been set to fixed values from literature [30].

## Results and Discussion

**Figure 1:**
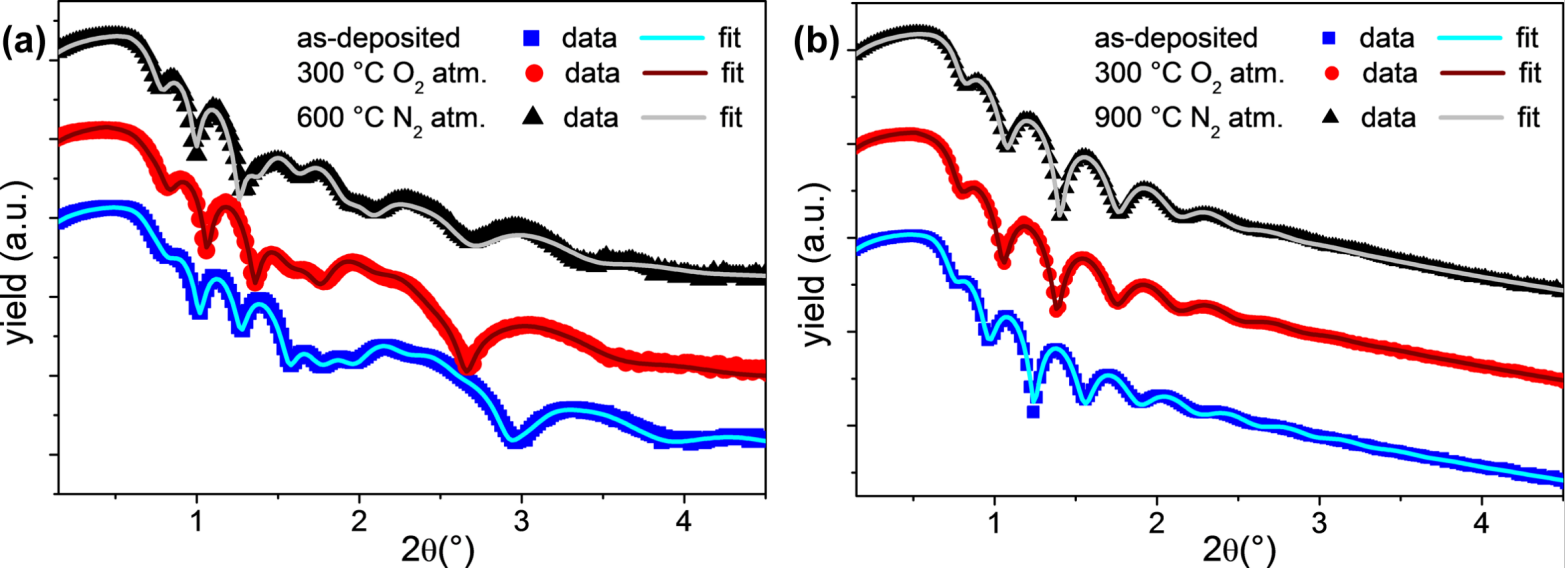
XRR experimental curves and data fitting of the (a) CeO_2_/TiN and (b) CeO_2_/Si: as deposited (blue symbols), annealed at 300 °C in O_2_ atmosphere (red symbols) and annealed at 600 °C or 900 °C in N_2_ atmosphere (black symbols). The fitting curves are also shown.

First, we performed the XRR analysis on CeO_2_ films deposited on both TiN and Si to extract thickness, roughness and density of the films. By considering the evolution of these film parameters, the technique can be used to retrieve insights on the stability of the films after each annealing treatment. Thus, the analysis has been performed in the as-deposited and in the annealed samples (annealed at 300 °C in O_2_ atmosphere for both substrates, annealed at 600 °C during diffraction acquisition in N_2_ atmosphere for CeO_2_/TiN and annealed at 900 °C during diffraction acquisition in N_2_ atmosphere for CeO_2_/Si). XRR data are resumed in Figure 1a for CeO_2_/TiN and Figure 1b for CeO_2_/Si.

By fitting the XRR profiles of the as-deposited samples and the samples annealed at 300 °C we found out that this mild annealing does not induce any significant change in the film thickness, roughness and density of CeO_2_. However, it is effective in the removal of the humidity absorbed at the surface, detected in the as-deposited samples only [8]. On the other side, when comparing the XRR profiles of the as-deposited samples with those annealed at high temperatures, we obtained different results, as shown by the fitting parameters reported in Table 1. In particular, we deduced an evolution of the composition of TiN, correctly simulated in a two-layer model, where the properties of one layer are compatible with highly oxidized TiN (TiNO*_x_*). CeO_2_ on Si showed no changes in the main features upon annealing up to 900 °C. Despite the slight increase of density upon annealing, the CeO_2_ layer maintains low roughness at the surface and at the interface with the substrate; the 7 nm thick interlayer included in the model accounts for the suspected further oxidation of the silicon substrate during annealing.

**Table 1:** Summary of the main parameters extracted from fitting XRR and XRD data of CeO_2_/TiN after annealing at 600 °C and CeO_2_/Si after annealing at 900 °C.

sample ID	thickness (nm)	interface roughness (nm)	surface roughness (nm)	electron density (e/Å^3^)	crystallite size (nm)	
					(111)	(200)

CeO_2_ on TiN (600 °C)	22.9 ± 0.4	1.1 ± 0.1	1.9 ± 0.1	1.61 ± 0.05	9.2 ± 0.5	12.5 ± 0.5
CeO_2_ on Si (900 °C)	21.5 ± 0.4	0.4 ± 0.1	1.5 ± 0.1	1.77 ± 0.05	13.5 ± 0.5	13.9 ± 0.5

**Figure 2:**
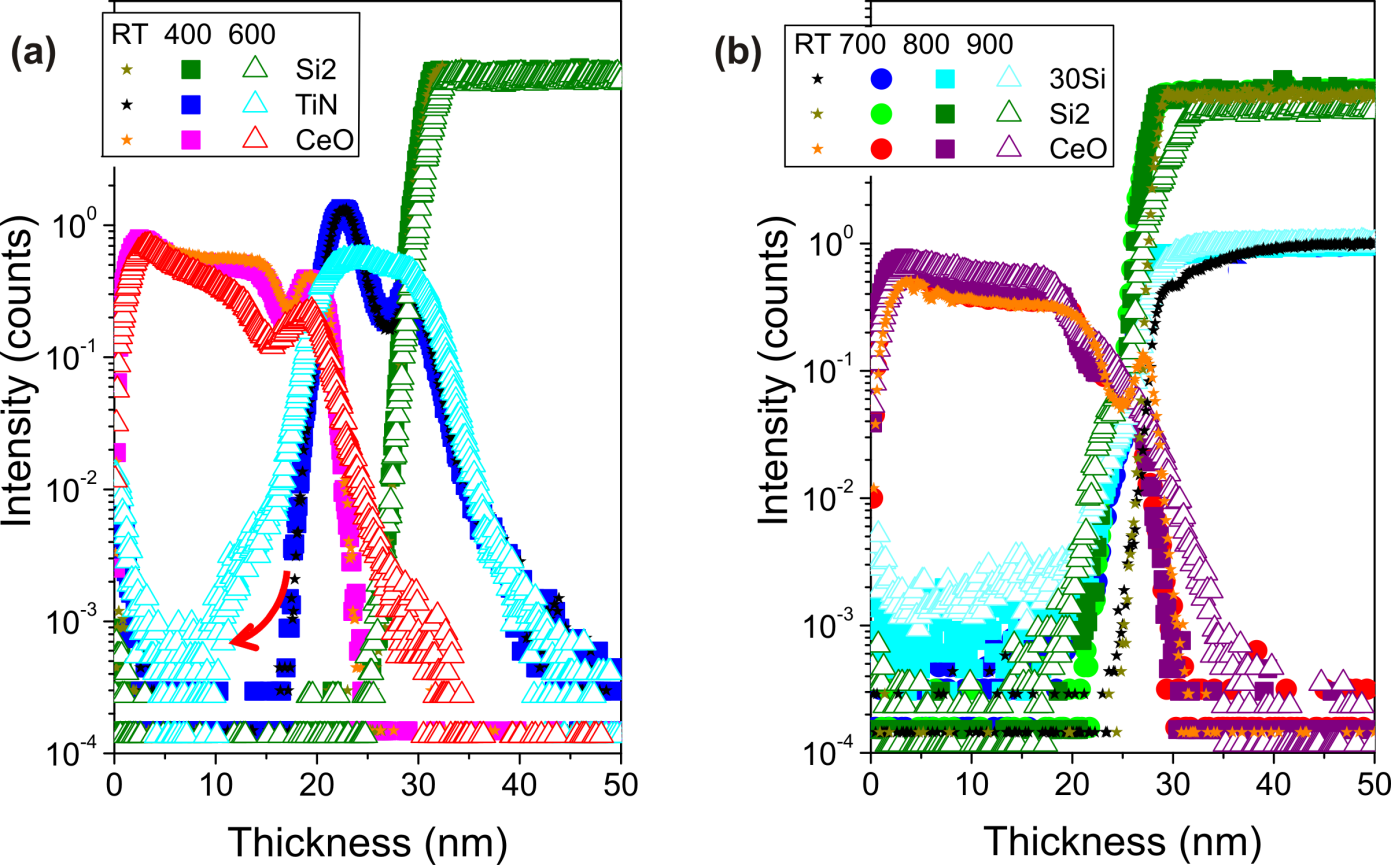
ToF-SIMS depth profiles of (a) CeO_2_/TiN as-deposited (RT) and annealed at 400 and 600 °C in N_2_ and (b) CeO_2_/Si as-deposited (RT) and annealed at 700, 800 and 900 °C in N_2_.

ToF-SIMS depth profiles collected from the samples annealed at high temperature confirm the XRR findings. To further clarify the sample response to the annealing treatments, we collected depth profiles from samples annealed also at low temperatures (Figure 2). In particular, Figure 2a reports the comparison among the profiles of CeO_2_/TiN, as-deposited (stars), annealed at 400 °C (squares) and 600 °C (triangles). In the as-deposited sample, the CeO signal is flat across the film thickness indicating the compositional homogeneity of the film, moreover the CeO_2_/TiN interface appears sharp and well defined. At 400 °C the film/substrate interface is still defined as in the as-deposited sample, while at 600 °C the interface appears broader. Further, we detect the evidence of TiN oxidation over almost its entire thickness. On the other side, no variation of the CeO profile is detectable apart from a tail broadening at increasing sputtering times, further evidence of CeO_2_/TiN interface instability. We note here that the instability of the CeO_2_/TiN interface imposes a severe limit in the integration of CeO_2_ films upon TiN in processes requiring exposure to temperatures above 600 °C after CeO_2_ deposition, as is required in many conventional CMOS processes. Because of such severe instability, we limited our considerations on this type of samples to this temperature.

In Figure 2b we compare ToF-SIMS profiles of CeO_2_ on Si as-deposited (star symbols), and annealed at 700 (circles), 800 (squares) and 900 °C (triangles). These progressive annealing treatments allow us to observe the evolution of the SiO_2_ interfacial layer thickness in between CeO_2_ and silicon substrate. It increases from ca. 2 nm to ca. 5 nm due to the onset of (oxygen) interdiffusion at the film/substrate interface. However, there is no change in the CeO profiles with temperature, and the broadening of the interface is limited even at 900 °C. Moreover, the C signal goes below the detectable limit and also the signal connected with OH^−^ content progressively decreases (not shown). This demonstrates that ceria film deposited on SiO_2_/Si substrate can sustain annealing at high temperatures up to 900 °C without major concerns regarding the onset of interdiffusion and interface stability.

**Figure 3:**
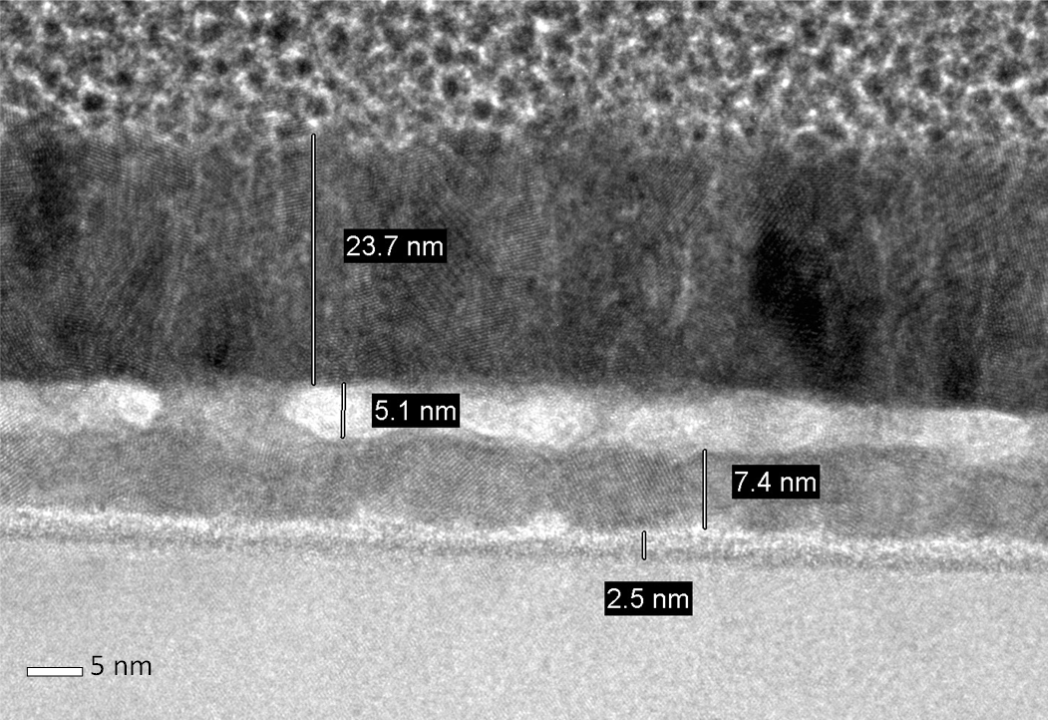
TEM image (cross-sectional view) of CeO_2_ on TiN annealed at 600 °C.

The cross-sectional view TEM image of CeO_2_/TiN (Figure 3) confirms ToF-SIMS observations about the interfaces, showing the broadening of the CeO_2_/TiN interface and the appearance of a thick (ca. 5 nm) interlayer of oxidized TiN or of mixed composition (Ti–N–O). We can further observe that the CeO_2_ layer on TiN deos not appear well ordered, as only in small regions of the film is possible to clearly recognize ordered planes, suggesting an incomplete crystallization.

**Figure 4:**
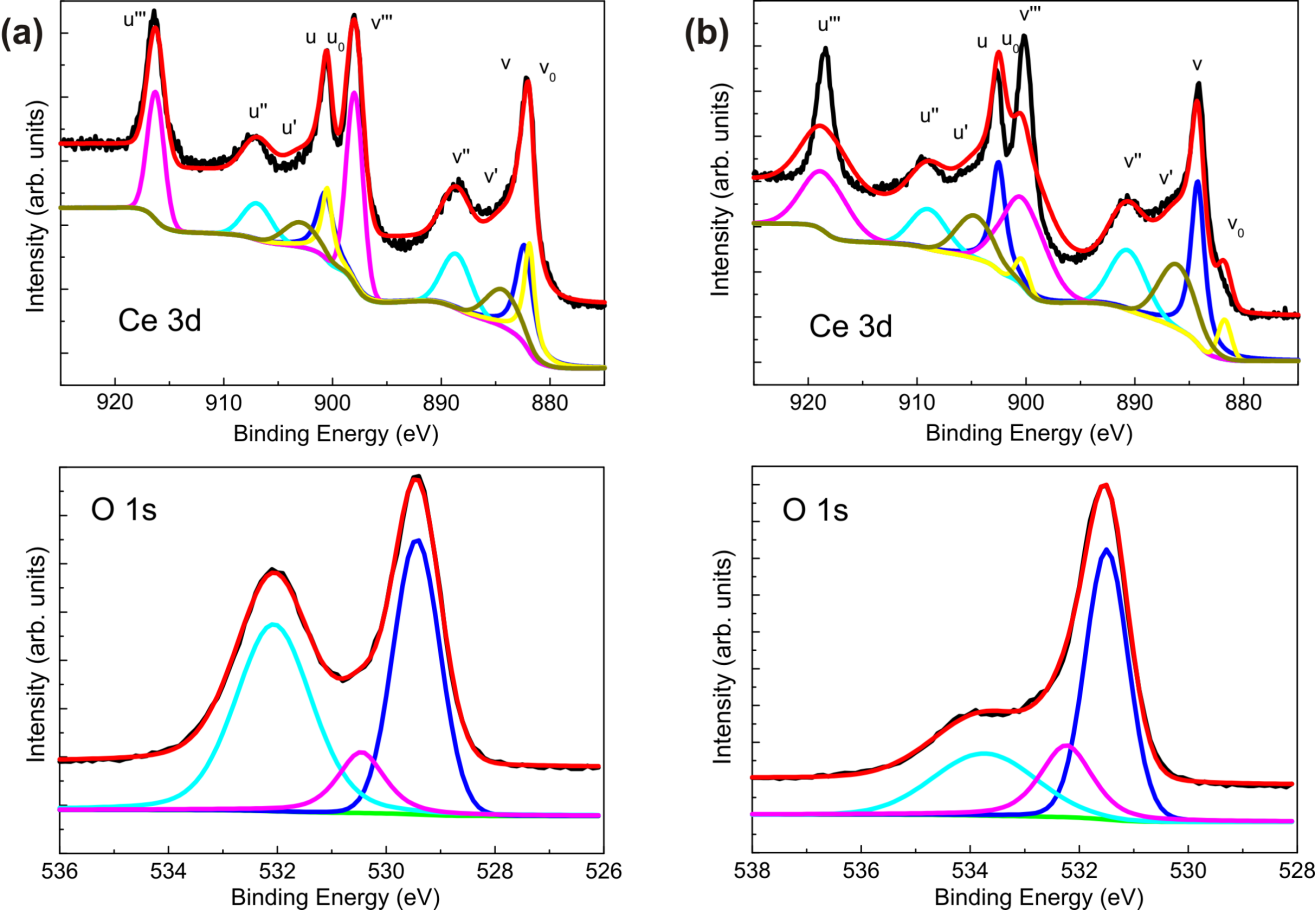
XPS spectra (both experimental and fitting curves) collected at 45° take-off angle of (a) CeO_2_/TiN after annealing at 600 °C and (b) CeO_2_/Si after annealing at 900 °C. The Ce 3d peak is shown in the upper panels and the O 1s peak in the lower panels. Fitting components are also shown.

XPS analysis of the samples annealed at high temperature gives us insights on the off-stoichiometry of the oxide layers induced by the thermal treatment. The technique probes the outermost part of the CeO_2_ layers and thus can be influenced by the interaction with the atmosphere. However, the estimation of the Ce^3+^/Ce^4+^ ratio can still be considered an indication of the chemical composition of the whole oxide layer. Figure 4 shows the Ce 3d (upper panel) and O 1s (lower panel) XPS spectra of the CeO_2_/TiN sample annealed at 600 °C (Figure 4a) and of the CeO_2_/Si sample annealed at 900 °C (Figure 4b). The Ce 3d peak is characterized by the presence of six distinct satellites, which have been labelled in the figure in accordance with the literature [31–33]. The “v” labels refer to the 3d_5/2_ spin–orbit split component, while the “u” labels mark the 3d_3/2_ signals. The figures also show the fit obtained from the deconvolution of the spectra into the different components, similarly to that reported in [6,9–10,13]. In Table 2, we reported the binding energies and relative peak areas of only “v” components for convenience. From the area of the different components of the Ce 3d spectrum (Table 2) we estimate the relative concentrations of Ce^3+^ and Ce^4+^ [25]. Similarly, considering the O 1s region, three components are identified and, from low to high binding energy, assigned to oxygen bonded to Ce and two components for adsorbed OH/CO*_x_*^2−^ complexes from the environment [34–35]. The resulting Ce^3+^ concentration in annealed CeO_2_/TiN is 29.3%, slightly higher than in the as-deposited sample. However, in terms of the chemical composition, the CeO_2_ film on TiN remains unaltered by the annealing treatment up to 600 °C. In the CeO_2_/Si sample the resulting Ce^3+^ concentration after annealing at 900 °C is 31.7%, much higher than in the as-deposited sample (where the concentration was 21.8%). This means that after high-temperature annealing CeO_2_ is chemically changed, probably due to the loss of oxygen and to the formation of CeO_2−δ_.

**Table 2:** Summary of peaks assignment, position and relative area of Ce3d_3/2_ peaks with different final oxidation states for CeO_2_ on TiN annealed at 600 °C (upper part) and CeO_2_ on Si annealed at 900 °C (lower part).

sample ID	Ce species	binding energy (eV)	relative area (%)

CeO_2_ on TiN annealed at 600 °C	Ce^3+^ (v_0_)	881.9	17.2
	Ce^4+^ (v)	882.4	22.3
	Ce^3+^ (v')	884.3	12.5
	Ce^4+^ (v'')	888.6	15.8
	Ce^4+^ (v''')	897.9	32.2

CeO_2_ on Si annealed at 900 °C	Ce^3+^ (v_0_)	879.6	0.9
	Ce^4+^ (v)	881.7	22.6
	Ce^3+^ (v')	883.2	30.8
	Ce^4+^ (v'')	888.4	14.8
	Ce^4+^ (v''')	897.5	30.9

**Figure 5:**
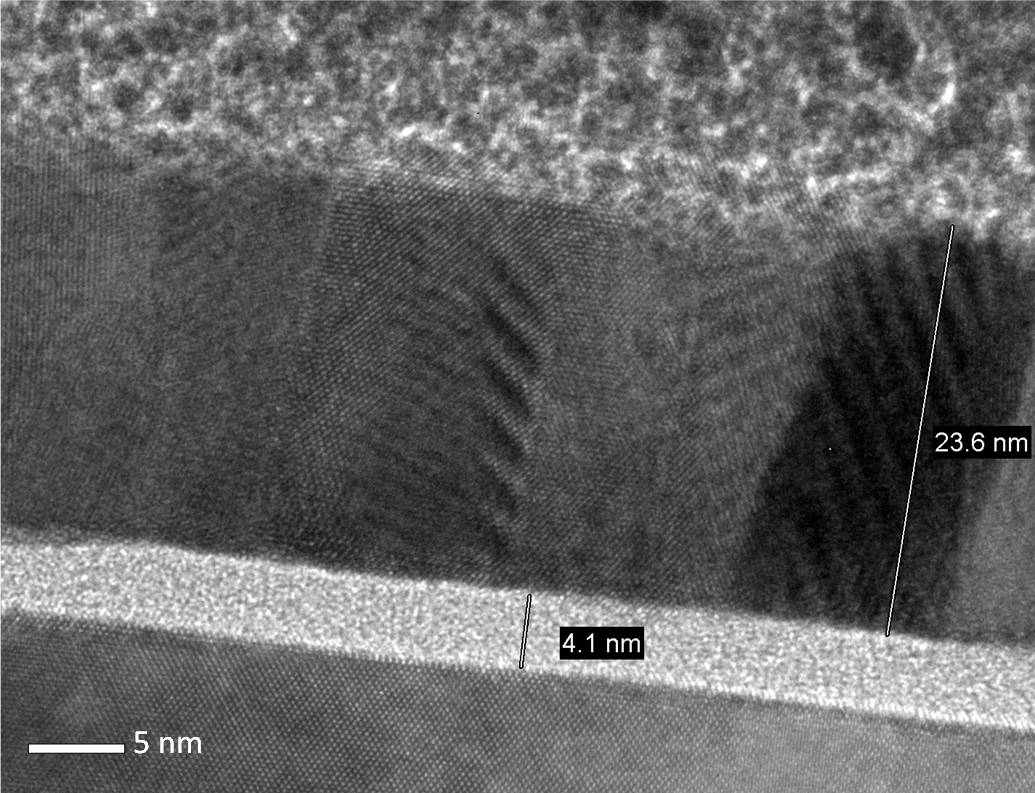
TEM image (cross-sectional view) of CeO_2_ on Si annealed at 900 °C.

The cross-sectional view TEM image of CeO_2_/Si after 900 °C (Figure 5) is very significant in indicating the changes in the film structure induced by the annealing. In addition to a well-defined CeO_2_/Si interface and a native SiO_2_ layer, we can clearly detect a columnar CeO_2_ structure with highly defined planes, even if it is not possible to identify which family of planes they belong to. This is a clear indication that the thermal treatment at 900 °C effectively promotes further crystallization of CeO_2_.

**Figure 6:**
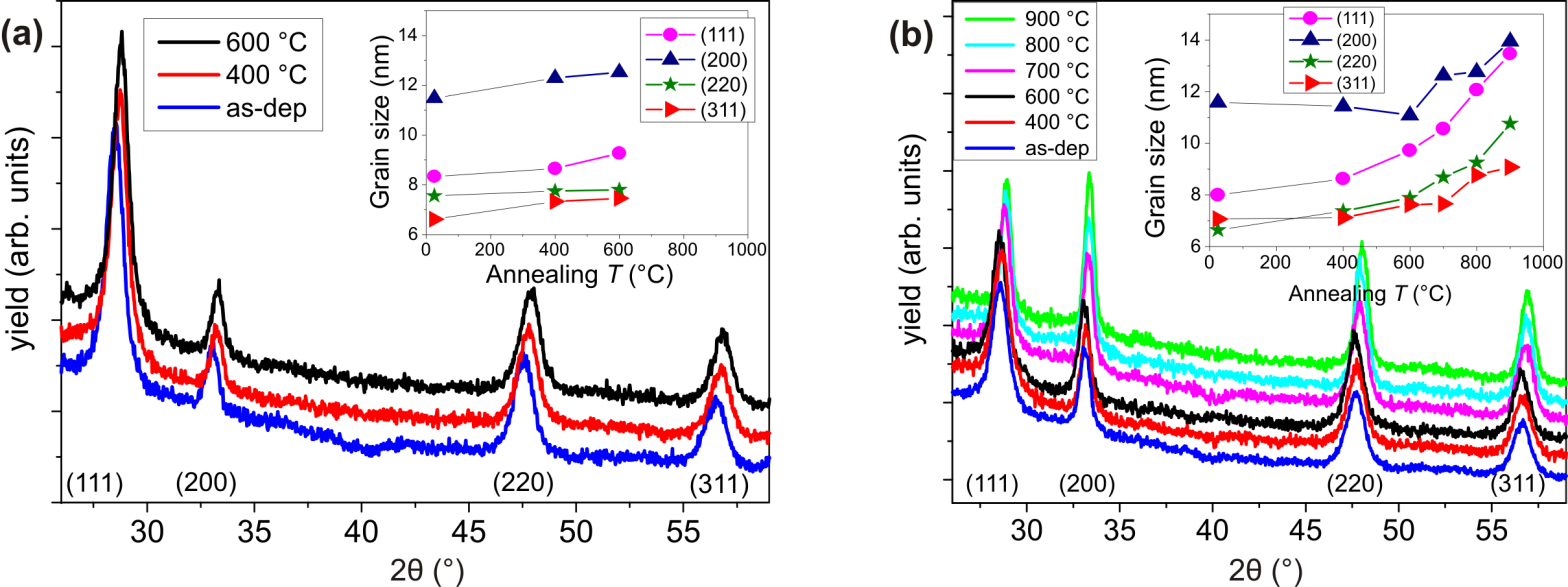
GIXRD profiles of (a) CeO_2_/TiN and (b) CeO_2_/Si as-deposited and in situ annealed during acquisition. In both panels, the inset shows the crystallite size calculated for each plane direction.

This consideration becomes consistent by checking the diffraction pattern at progressively higher temperature of the ceria films grown on the two different substrates. Indeed, in this way we can show the evolution of the film structure and the phase purity during the thermal treatment. In Figure 6a we report the diffraction pattern at grazing incidence from CeO_2_/TiN as deposited (red line), annealed at 400 °C (blue line) and annealed at 600 °C (black line). Here, we see that no evident change in the peaks intensity and FWHM is induced upon annealing, neither there is the formation of other crystalline phases. We also calculated the crystallite size by Scherrer analysis for each family of planes (111), (200), (220) and (311) as a function of the annealing temperature (inset of Figure 6a). Even at 600 °C the increase in crystallite size is almost negligible (see Table 1), indicating that the thermal budget supplied to the system is not enough to promote crystallization.

A different behavior is observed from diffraction patterns at grazing incidence of CeO_2_/Si as-deposited (blue line), and annealed at 400 °C (red line), 600 °C (black line), 700 °C (pink line), 800 °C (light blue line) and 900 °C (green line) reported in Figure 6b. In this case, we observe a progressive increase in the intensity and narrowing of the peaks with temperature, especially at 800–900 °C, sign of an increase of grain number and size. In particular, we notice that after annealing at 900 °C the (111) and (200) peaks have the same intensity. This is even clearer looking at the inset of Figure 6b: After annealing at 900 °C, the (111) and (200) crystallites have equivalent size, Further, we notice that after annealing at 900 °C the (111) and (200) peaks have the same intensity, which hints to a preferential crystallographic orientation of the film.

**Figure 7:**
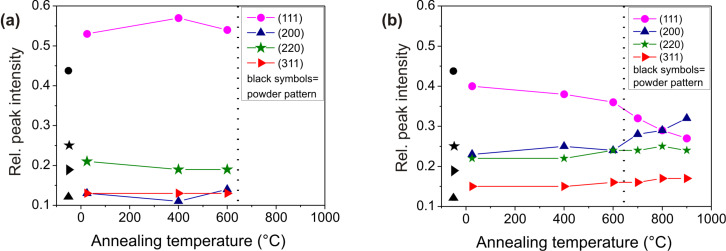
Fraction of the crystalline planes oriented in each respective crystalline direction as a function of annealing temperature for (a) CeO_2_/TiN and (b) CeO_2_/Si.

As a further hint to the presence of a preferential orientation, we plotted the relative intensities of the main peaks as a function of the annealing temperature (Figure 7). Even if the intensities of reflections are not a direct measure of the corresponding crystallographic orientations, we can use these relative intensities as indication of a preferential orientation by comparing them with the relative intensities of a randomly oriented polycrystalline material (black symbols). In the case of CeO_2_ deposited on TiN, the prevalence of the (111) orientation and its preservation upon annealing is immediately evident. Considering CeO_2_ deposited on Si, annealing up to 600 °C induces no change in peak intensity (as expected) compared to the as-deposited sample. However, there is an evident change in the relative intensity of CeO_2_ peaks at higher temperatures. The already existing preferential orientation of the as-deposited CeO_2_/Si sample is enhanced after high temperature annealing: In the film there is an increased number of grains with (200) orientation in addition to those with (111) orientation.

**Figure 8:**
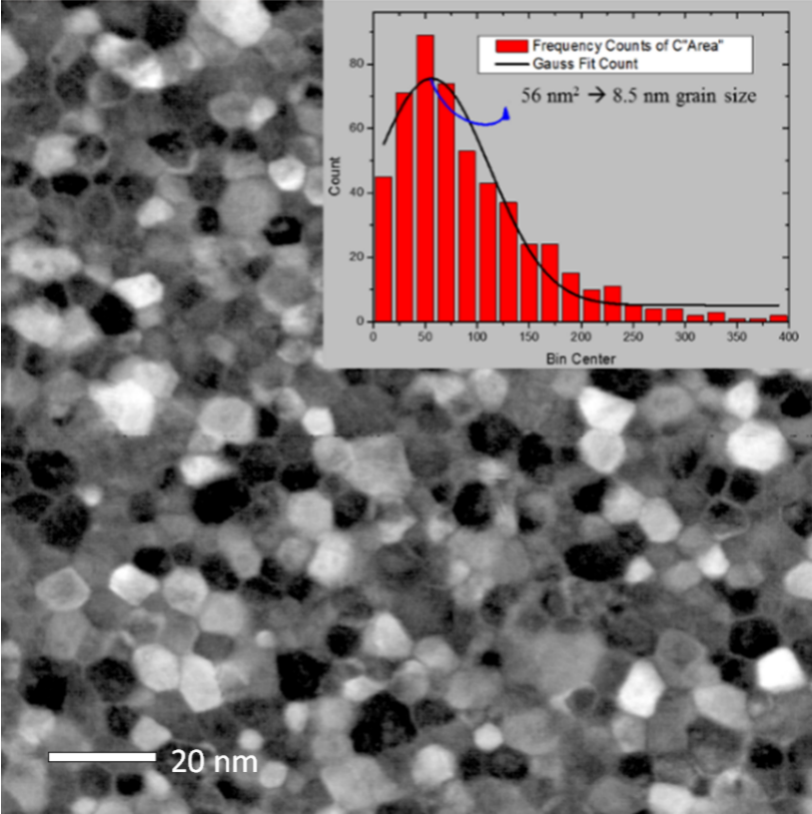
TEM image (plan-view) of CeO_2_ on Si annealed at 900 °C. Inset: histogram showing the grain size distribution.

**Figure 9:**
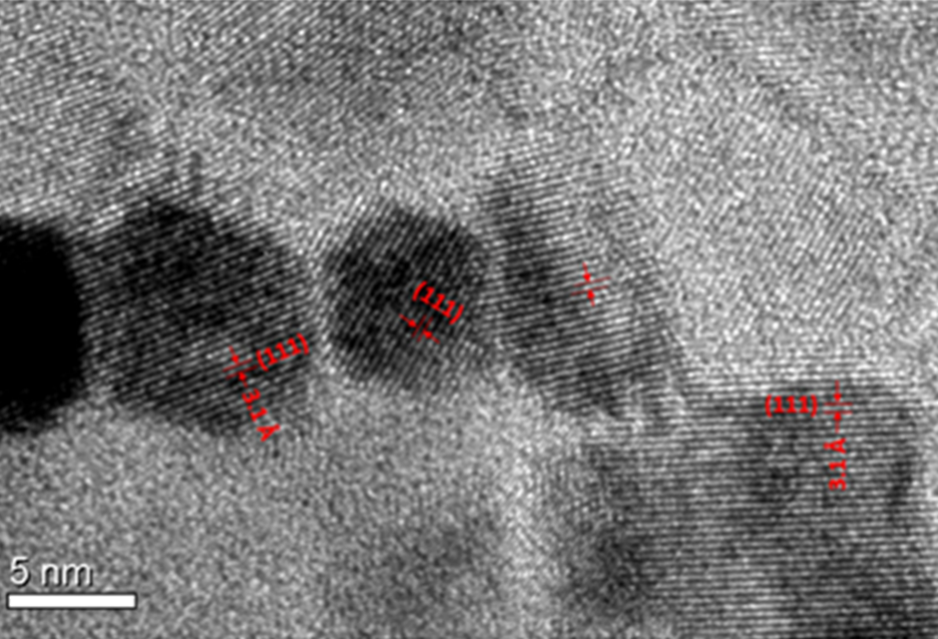
High-resolution TEM image (plan-view) of CeO_2_ on Si annealed at 900 °C. The lattice fringes of (111) CeO_2_ planes are also shown.

The plan-view TEM image in dark field of the CeO_2_/Si sample (Figure 8) is very impressive in revealing the compact and spherical shape of the grains formed after treatment at 900 °C, further confirming its full crystallization evidenced by cross-sectional view TEM in Figure 5. From this image, the crystallite sizes are estimated to be in the range of 8–12 nm (see the histogram in the inset of Figure 8). This is in line with the value obtained from GIXRD. The high-resolution TEM image (Figure 9) shows CeO_2_ grains and allows for the identification of the (111) planes, having a plane distance of 0.31 nm, in agreement with the value obtained from GIXRD.

Summarizing the whole set of data collected, we can conclude that CeO_2_ is stable up to 600 °C with no annealing effects on its structure or chemical composition. Moreover, annealing (at 300 °C in O_2_) helps in the removal of the adsorbed moisture and in reducing residual contaminants, such as carbon, from the deposition process. However, the annealing induces severe oxidation on TiN with a broadening of the interface with CeO_2_ and potential local mixing, which limit its integration in a technological process. On the contrary, the high stability of the Si substrate (apart from its oxidation at the interface) allows to increase the annealing temperature up to 900 °C, which supplies to the system the energy budget required for the complete crystallization. In addition, we evidence that the ceria film grows with a different preferential orientation depending on the substrate, a difference that is enhanced when the film is completely crystallized.

This behavior can be tentatively set using the model already reported for CeO_2_ grown by MOCVD at different temperatures and on different substrates [36], adjusting the interpretation to our ALD growth. Generally, from atomistic simulation it has been estimated that the energy stabilities of the CeO_2_ surfaces follows the order of (111) > (110) > (100) [37], since the formation on <100> surface of dipoles [38]. However, the preferential orientation of grains in the film grown on different substrates depends on the substrate surface, on the roughness and on the balance (or imbalance) between the intrinsic elastic stress and the surface energy. Above a certain critical film thickness, the surface energy which favors the (111) orientation does not contribute anymore, while the elastic energy, which can be better stored in grains with (200) orientation, correspondingly increases. Thus, grains with the latter orientation are formed. At the free surface of the CeO_2_ layer, the situation changes again, and it can be energetically favorable to expose the (111) planes rather than the (200) planes, since the latter is considered unstable due to the dipole formed perpendicularly to it [39]. Indeed, the growth of grains along the <100> direction capped by pyramids with the four triangular faces of (111) planes [40] is commonly observed. In our ALD-grown samples, we have to consider factors that specifically depend on the deposition itself. Due to the ALD deposition temperature (250 °C), it is possible that the Ce(thd)_4_ molecules do not effectively diffuse onto the substrate surface and the surface coverage on the TiN or Si substrates remains strictly connected to the surface roughness, which is higher on the TiN surface (compared to Si). We should keep in mind that the sticking coefficient of Ce(thd)_4_ depends also on the nature of the surface. Metallic surfaces offer less centers of nucleation for diketonate molecules, implying a further reduction of the complete surface coverage and leaving space between Ce atoms to be occupied by O atoms during the subsequent O_3_ pulse. The alternating presence of Ce and O atoms promotes the formation of (111) planes. Only after this first stage, the elastic energy starts to be more effective and the constraint due to the substrate surface less strict, inducing the formation of large (200)-oriented grains. When the deposition is on Si, the precursors encounter a layer of native SiO_2_. Since the Ce atoms tend to combine with oxygen, it is easier to form a Ce layer, which completely covers the substrate, and then to bond oxygen atoms to Ce with the next ALD pulse. Alternating planes of Ce and O atoms are (200) planes. This explains why in CeO_2_/Si we observe not only large (200)-oriented grains, but also a preferential (200) orientation. Upon annealing at sufficiently high temperature, CeO_2_ atoms acquire sufficient mobility to accommodate themselves minimizing the total energy, favoring the formation of (111) surface. Grains with both (111) and (200) orientations grow in dimension while the accumulation of elastic energy further enhances the (200) texturing.

## Conclusion

In conclusion, polycrystalline CeO_2_ films prepared via ALD with cubic fluorite structure have been studied after annealing at 300 °C in O_2_ atmosphere and after high-temperature annealing (600 °C for CeO_2_/TiN and 900 °C for CeO_2_/Si). In CeO_2_/Si the cubic phase and the preferential orientation detected in as-deposited films are maintained up to 900°C, with a further enhancement of (200) planes upon annealing. This is a strong indication that the texturing is an intrinsic property of the system, due to the contribution of different energy components depending on the structure and composition of the substrate, and not a metastable condition related to the growth conditions. However, the integration of CeO_2_ on TiN is limited to 600 °C due to a broadening of the interface and the chemical instability of TiN, although CeO_2_ does not show any relevant change. Ceria deposition on metallic substrates is scarcely explored, especially regarding film thickness, and there are no reports on the consequences of thermal treatments of this type of stack. In this respect, our work is relevant because it underlines the feasibility of integrating CeO_2_ in technological processes, such as back-end CMOS processes, at the same time uncovering the limitations. In particular, we demonstrated that the ratio of Ce^3+^/Ce^4+^ is not heavily affected by annealing treatments, an argument of relevance in perspective of the integration of CeO_2_ as dielectric material in capacitors. In this respect, the electrical characterization of our CeO_2_ films in a metal–insulator–metal (MIM) capacitor would be useful to study the electrical behavior of CeO_2_. However, this is beyond the scope of our study.

On a more fundamental point, the study of the first deposition steps of CeO_2_ by thermal ALD upon different substrates is required to fully elucidate the mechanism of the growth and nucleation of CeO_2_, and possibly validate our proposed phenomenological model. A possible approach would be the study by dedicated in situ ALD growth at beamlines using synchrotron radiation to probe the chemistry and local organizations at the atomic level.

Finally, we believe that our results may be useful in terms of enabling future materials and device development, with the aim of controlling key film parameter such as texturing when relevant for technological applications.
